# Editorial: Stress-responsive microbiome of horticultural plants: diversity, functions, and application prospects

**DOI:** 10.3389/fmicb.2026.1923431

**Published:** 2026-08-24

**Authors:** Mengjun Huang, Xiang Tao, Xinrong Ma, Yinghong Gu, Yusong Jiang

**Affiliations:** 1 Research Institute for Special Plants, Chongqing University of Arts and Sciences, Chongqing, China; 2 Key Laboratory of Land Resources Evaluation and Monitoring in Southwest, Ministry of Education, College of Life Sciences, Sichuan Normal University, Chengdu, China; 3 Chengdu Institute of Biology, Chinese Academy of Sciences, Chengdu, China; 4 Department of Biochemistry, University of Oxford, Oxford, United Kingdom

**Keywords:** application, cross-kingdom interactions, horticultural plants, microbial diversity, stress response

Horticultural plants are continually subjected to a wide array of abiotic and biotic stressors. The stress-responsive microbiome, a dynamic, taxonomically diverse assemblage of bacteria, fungi, and archaea, contributes critically to plant resilience by facilitating nutrient acquisition, modulating phytohormone homeostasis, and suppressing pathogenic microorganisms. Recent advances in multi-omics technologies and bioinformatics have significantly enhanced our ability to decipher host-microbe interactions, thereby supporting the development of sustainable agriculture.

Significant advances have been achieved in elucidating the intricate interactions between plants and microbes. These interactions hold considerable promise for plant health, primarily through the enhancement of plant-growth-promoting rhizobacteria and/or the suppression of phytopathogens. However, our understanding of the spatiotemporal dynamics, functional specificity, molecular mechanisms, and key environmental and host-derived determinants of microbiomes remains incomplete. To address these, we proposed the Research Topic entitled “*Stress-responsive microbiome of horticultural plants: diversity, functions, and application prospects*,” which integrates current findings, critical insights, and forward-looking perspectives on microbial community responses to abiotic and biotic stressors, the foundations of microbiome-mediated resilience, and strategies for microbiome-informed horticultural management. This Research Topic comprises four original contributions as below.

## Root-associated microbial community of resurrection plant

Plants modulate microbial diversity and ecosystem function through phytohormonal signaling and defense responses. Asatulloev et al. investigated the root-associated microbial communities of the resurrection plant *Oreocharis mileensis* across five populations. Microbial composition was significantly influenced by environmental compartments, with moderate drought-induced shifts observed alongside a stable core microbiome. Conserved phyla were present across populations, although genus-level taxa varied, suggesting host genotype-driven niche specialization. Drought increased bacterial alpha diversity but reduced beta diversity, indicating homogenization driven by stress-tolerant taxa. Functional profiling revealed compartment-specific metabolic profiles and enriched stress-response pathways, positioning the *O. mileensis* microbiome as a resilient system that supports plant survival under stress. This study advances our understanding of microbial assembly in resurrection plants and identifies candidate microbes for engineered microbiome interventions aimed at enhancing crop stress tolerance.

## Rhizosphere microbes of tea plants contribute to differences in tea quality

The zonal “rock flavor” of Wuyi rock tea may be linked to rhizosphere microbes. One study compared microbial communities and tea quality across three rocky zones: the authentic rocky (ZY) zone, the semi-rock (BY) zone, and the continental (ZC) zone. The results indicate that ZY contains higher concentrations of key quality-associated components, such as catechin, theanine, and caffeine, than both BY and ZC. Soil physicochemical analyses revealed that ZY has the highest levels of available nitrogen and phosphorus, while ZC had a greater organic matter content. Microbial community analysis showed that ZY harbored the greatest microbial functional diversity but exhibited the lowest network complexity, suggesting that random processes predominate in community assembly. Machine learning identified *Obscuribacteraceae* and *Verrucariaceae* as critical families positively correlated with tea quality indices; these families were also enriched in photosynthesis and lichenization pathways. Overall, these findings elucidate the role of soil microbial communities in shaping tea quality and highlight their potential for precision management in tea cultivation (Jia et al.).

## Fertilization alters rhizosphere microbial communities to affect tea plant growth

Fertilization is a key agronomic practice, yet its effects on the rhizosphere microbial communities of tea plants remain unclear. One contribution demonstrated that fertilization strategies significantly influence the diversity and functional dynamics of rhizosphere soil microbial communities associated with tea plants. Of the three fertilization treatments evaluated [100% chemical fertilizer (CF), 50% chemical fertilizer combined with 50% organic fertilizer (COF), and 100% organic fertilizer (OF)], the COF treatment markedly enhanced tea plant growth, as evidenced by increases in chlorophyll content, leaf area, and hundred-bud weight. Microbial assessments revealed that the COF treatment significantly boosted microbial biomass carbon and phosphorus, whereas the CF treatment was most effective at increasing microbial biomass nitrogen. Furthermore, high-throughput sequencing highlighted that the COF treatment altered bacterial community structure, promoted beneficial microorganisms, and enhanced soil functions related to lichenization while reducing fermentation-related functions. These findings underscore the synergistic benefits of combined fertilization for both soil health and tea growth, providing insights for optimizing fertilization strategies in tea plantations (Zhang et al.).

## Development of RAA-LFD assay for the diagnosis of powdery scab in potatoes

One article presented a significant advancement in the diagnosis of potato powdery scab, a soilborne disease caused by *Spongospora subterranea*. A species-specific genomic DNA fragment of 2,494 bp was identified through comparative genomics, which contributed to the development of a polymerase chain reaction (PCR) assay and a recombinase-aided amplification-lateral flow dipstick (RAA-LFD) assay for pathogen detection. The detection sensitivity of the PCR primers reached as low as 10.3 copies, while the RAA-LFD assay exhibited superior performance, particularly in soil samples, with a detection limit as low as 25.1 copies for the RAA primer and probe set. Notably, the RAA-LFD method demonstrated rapid and reliable detection across all tested tubers and soil samples, positioning it as a promising field-deployable diagnostic tool for the effective management of potato powdery scab. This innovative approach not only enhances pathogen identification but also holds critical implications for improving potato management practices (Yang et al.).

